# Dual proteomics of infected macrophages reveal bacterial and host players involved in the *Francisella* intracellular life cycle and cell to cell dissemination by merocytophagy

**DOI:** 10.1038/s41598-024-58261-x

**Published:** 2024-04-02

**Authors:** Héloïse Rytter, Kevin Roger, Cerina Chhuon, Xiongqi Ding, Mathieu Coureuil, Anne Jamet, Thomas Henry, Ida Chiara Guerrera, Alain Charbit

**Affiliations:** 1 Université Paris CitéINSERM UMR-S1151, CNRS UMR-S8253Institut Necker Enfants Malades, 156-160 rue de Vaugirard, 75015 Paris, France; 2 INSERM U1151-CNRS UMR 8253, Team 7: Pathogénie des Infections Systémiques, 75015 Paris, France; 3 INSERM US24/CNRS UAR3633, Proteomic Platform Necker, UniversitéParis-Cité, Federative Research Structure Necker, Paris, France; 4 CIRI, Centre International de Recherche en Infectiologie, Université Lyon, Inserm, U1111, Université Claude Bernard Lyon 1, CNRS, UMR5308, Ecole Normale Supérieure de Lyon, 69007 Lyon, France

**Keywords:** Bacterial host response, Pathogens

## Abstract

Bacterial pathogens adapt and replicate within host cells, while host cells develop mechanisms to eliminate them. Using a dual proteomic approach, we characterized the intra-macrophage proteome of the facultative intracellular pathogen, *Francisella novicida*. More than 900 *Francisella* proteins were identified in infected macrophages after a 10-h infection. Biotin biosynthesis-related proteins were upregulated, emphasizing the role of biotin-associated genes in Francisella replication. Conversely, proteins encoded by the *Francisella* pathogenicity island (FPI) were downregulated, supporting the importance of the *F. tularensis* Type VI Secretion System for vacuole escape, not cytosolic replication. In the host cell, over 300 proteins showed differential expression among the 6200 identified during infection. The most upregulated host protein was cis-aconitate decarboxylase IRG1, known for itaconate production with antimicrobial properties in *Francisella*. Surprisingly, disrupting IRG1 expression did not impact *Francisella*’s intracellular life cycle, suggesting redundancy with other immune proteins or inclusion in larger complexes. Over-representation analysis highlighted cell–cell contact and actin polymerization in macrophage deregulated proteins. Using flow cytometry and live cell imaging, we demonstrated that merocytophagy involves diverse cell-to-cell contacts and actin polymerization-dependent processes. These findings lay the groundwork for further exploration of merocytophagy and its molecular mechanisms in future research.

Data are available via ProteomeXchange with identifier PXD035145.

## Introduction

*Francisella tularensis* is the causative agent of the zoonotic disease tularemia^1^. This facultative intracellular bacterial pathogen is able to infect numerous cell types but is thought to replicate and disseminate mainly in macrophages in vivo^2^. The four major subspecies (subsp) of *F. tularensis* currently listed are the subsps: *tularensis*, *holarctica, mediasiatica* and *novicida* (the latter is also called *F. novicida*). These subsp differ in their virulence and geographical origin^3^ but all cause a fulminant disease in mice that is similar to tularemia in humans^4^. Although *F. novicida* is rarely pathogenic in humans*,* its genome shares a high degree of nucleotide sequence conservation with the human pathogenic subsp *tularensis* and is thus widely used as a model to study highly virulent subsp.

*Francisella* virulence is tightly linked to its capacity to multiply exclusively in the cytosolic compartment of infected cells, and in particular in macrophages in vivo. We aimed here to identify new actors of intracellular cycle of *Francisella* and its interaction with the host. For this, we performed a proteomics analysis of infected macrophages to explore both cellular as well as bacterial actors of the infection of *Francisella.*

Proteomics is challenging because of the underrepresentation of pathogen-derived proteins compared to the host cell proteins and has never been attempted for *Francisella*^5,6^. Dual proteomics is a powerful approach allowing the simultaneous analysis of two or more proteomes, such as the host and pathogen proteomes in an infected cell^7,8^. We applied dual proteomics to study the macrophages infected with *Francisella* to obtain a more comprehensive view of the bacterial and host proteins involved in the *Francisella* intracellular life cycle. To optimize our coverage of bacterial proteins we combined enrichment of infected macrophages using cell sorting and high-resolution mass spectrometry. We showed that bacterial biotin biosynthesis pathway is necessary for *Francisella* during its intracellular cycle, and that the infection triggers the higher expression of proteins involved in inflammation and cell adhesion in the host cells. No single protein invalidation was sufficient to disrupt the intracellular cycle of *Francisella*, suggesting that larger complexes may be involved. A more global analysis of proteins deregulated in the host pointed to some are involved in regulation of cytoskeleton organization, regulation of cell shape but also in cell–cell adhesion suggesting rearrangement may favor cell–cell contact. We therefore focused on the dissemination of *Francisella.* Indeed, cytosolic pathogens, that notably include *Listeria monocytogenes, Burkholderia pseudomallei*, *Rickettsia* subsp and *Shigella flexneri,* are able to pass from an infected to an uninfected cell by forming actin tails that propel them into new cells. In contrast, *Francisella* is also capable to infect cells by using cell-to-cell contact, but actin-based motility was not observed. Instead, *Francisella* uses a process, coined merocytophagy, that is still poorly characterized^9^. Using live cell imaging and cytometry approaches, we demonstrated for the first time here that this process involves different types of cell-to-cell contacts that we called “cellular clustering” and " Kiss and run “. Following up the hypothesis driven by the proteomics results, we also showed that merocytophagy is actin polymerization-dependent processes.

This study is the first proteomics analysis of *Francisella* infection of macrophages, and contributes to elucidate the replication and dissemination of this facultative intracellular pathogen.

## Results

### Proteomics of infected macrophages

**Figure 1:**
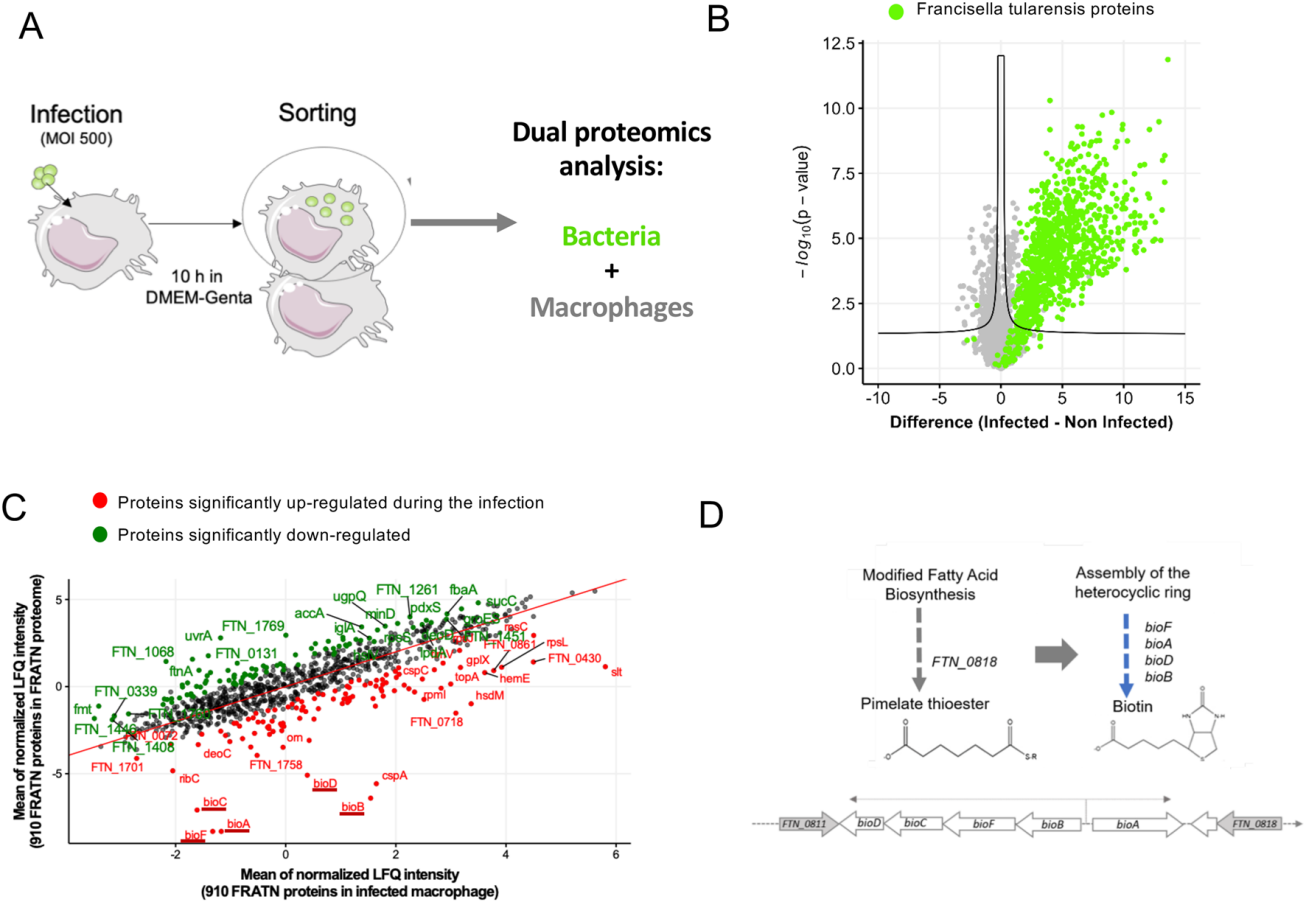
Bacterial proteomics from proteomic approach. (**A**) Model of dual proteomic approach of infected cells by *F. novicida.* After 10 h of infection, infected cells with GFP-expressing bacteria were sorted and collected for proteomic analysis. (**B**) Bacterial proteins of *Francisella* in infected macrophages identified by proteomic method (**C**) Scatter plot of mean LFQ intensity of FRATN proteins found in infected macrophage (x axis) and in FRATN proteome (y axis), respectively after width adjustment normalization. In green and in red are displayed the proteins significantly down- and up-regulated, respectively, when *Francisella* infects macrophage. Significance was assessed with Student’s t-test on Perseus v1.6.15 with s0 = 1 and permutation-based FDR 1% used as a truncation method. (**D**) Scheme of biosynthesis of biotin and operon of biotin genes.

In order to identify new actors of the intracellular cycle of *Francisella* we performed a proteomic analysis of macrophages infected with *F. novicida*. To enrich the cellular subset of infected macrophages, cells infected with GFP-expressing bacteria were sorted and collected for proteomic analysis (see Experimental procedures, and Fig. 1A). Our aim was to identify proteins, both bacterial and cellular, whose expression may vary upon infection with *F. novicida and* that could participate to intracellular adaptation and bacterial transfer. For this, we first compared the proteome of *F. novicida* during its cycle in macrophages with the proteome of bacteria in planktonic condition. Second, we compared the proteome of non-infected macrophages to that of macrophages infected with *F. novicida*. Analyses were performed in murine macrophage-like cell line J774A.1 and in bone marrow-derived macrophages (BMMs) from C57BL/6 mice.

### Proteomic of bacteria

The proteome of *F. novicida* was analyzed after 10 h of infection. At this stage the bacteria had adapted to the cellular environment and undergone multiplication. We could identify 910 bacterial proteins in the infected macrophage (i.e. more than 50% of the estimated *F. novicida* proteome) (Fig. 1B; Table S1). Such coverage prompted us to compare the relative expression of the identified protein to their relative expression in bacteria grown in rich liquid culture (in Schaedler K3 medium). The analysis of bacteria from 10 h culture in K3 (3 replicates) allowed to quantify 1567 proteins, in line of what we previously showed^10,11^.

Of the 910 bacterial proteins identified in the infected macrophages, 908 were also found and quantified in the bacterial proteome from culture (Table S2). The only two proteins that were identified exclusively in bacteria from infected macrophages were adenosylmethionine-8-amino-7-oxononanoate aminotransferase (BioA, FTN_0816_) and 8-amino-7-oxononanoate synthase (BioF) and they were included in the differential analysis, as they were likely overexpressed in the intracellular *Francisella* proteome. We focused on the relative quantification of the 910 proteins in the two bacterial samples. In order to directly compare the two proteomes, we first normalized the two data sets by alignment of the intensity distributions. We then performed a correlation analysis to identify bacterial proteins which are deregulated specifically during infection, and highlighted proteins that were significantly differential according to *t* test (FDR = 1%, S0 = 1) (Fig. 1C). Although the expression of the majority of proteins did not vary between the two conditions, we could observe that BioA, B, C, D, F and FTN_0718 (biotin lipoyl) were all increased in the bacteria engaged in the macrophage infection (Fig. 1C,D, Table S3). These 4 proteins belong to the biotin biosynthesis pathway in line with previous studies that have shown that biotin is essential for promoting rapid escape during the short time that the bacterium is in the phagosome and for its intracellular multiplication in the cytosol^12–14^. We confirmed here that biotin, and biotin-associated genes, play important role during replication phase of *Francisella*. Of note, most of the detected proteins encoded by the *Francisella* pathogenicity island (FPI) are downregulated in macrophages at 10 h, including IglA to IglD (FTN_1324 to FTN_1324), and IglE (FTN_1311), VgrG (FTN_1312), IglH (FTN_1315), DotU (FTN_1316) IglI (FTN_1317) as well as the effector opiA (FTN_0131). These data imply that *F. novicida* successfully acclimated to the macrophage cytosol in which the FPI-encoded T6SS (required for the early phagosomal escape) appears dispensable for replication^15,16^. Another bacterial protein that exhibited overexpression in infected macrophages is CspA, (cold shock protein A) which is believed to be associated with stress resistance^17^. The protein Slt (soluble lytic transglycosylase) was also upregulated in macrophages. This protein plays a role in intracellular growth and immune suppression^18^. In summary, these analyses suggest that *Francisella* overexpresses specific proteins that contribute to its adaptation and survival within host cells.

### Proteomics of macrophages

**Figure 2:**
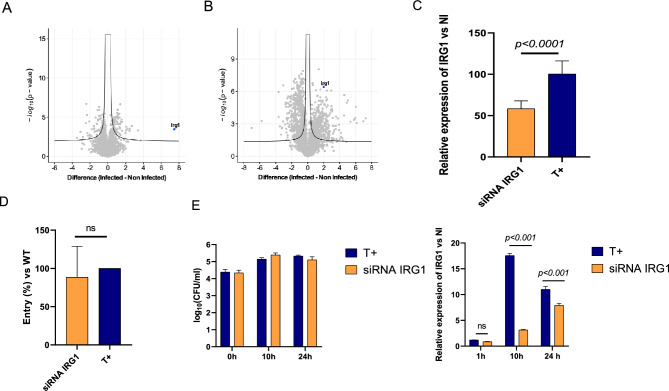
Proteomic of infected cells by *F. novicida* and role of IRG1 in entry and intracellular multiplication of *F. novicida*. (**A**) Volcano plot representing the statistical comparison of the protein LFQ intensities of infected J774A.1 (right) vs non infected cells. Volcano was established using S0 = 0.1, FDR = 0.01. The abscissa reports the fold change in logarithmic scale (difference), the ordinate the − log(pvalue). (**B**) Volcano Plots representing the statistical comparison of the proteome of infected BMMs vs non infected cells (S0 = 0.1, FDR = 0.01). the abscissa reports the fold change in logarithmic scale (difference), the ordinate the − log(pvalue). Percentage of (**C**) entry of *F. novicida* and (**D**) kinetic of intracellular multiplication was monitored in J774A.1 macrophages transfected with siRNA of IRG1 over a 24 h-period in DMEM supplemented with glucose and 10% of FBS, and compared to that in J774A.1 WT macrophages. The expression of IRG1 was analyzed at each point of kinetics by qRT PCR. P < 0.01 (as determined by ANOVA test).

Results from *J774A.1 macrophages*. We focused on the host response once *Francisella* is adapted to cellular environment and compared the infected to non-infected macrophages proteomes. Using the same LC-MSMS condition, over 6223 proteins could be quantified in the macrophages across samples (mean of 6183 proteins in non-infected cells and 6119 in infected cells), rendering the proteome of infected macrophage comparable its non-infected control (Fig. 2A, Table S1). After performing bacterial proteome subtraction and minor normalization we found 139 host cell proteins differentially expressed, 52 were upregulated and 87 downregulated upon infection with *Francisella.*

Among the host cell proteins differentially expressed, nine were upregulated more than 1.5-fold upon infection with *Francisella*. IRG1 was the main cellular protein that was upregulated upon infection (> 7-fold). This protein is encoded by the immune-responsive gene 1 (*irg1* also designated *acod1*). Remarkably, this gene is not expressed at the baseline state of macrophages and is strongly up-regulated in activated macrophages, making it the most highly induced genes (Fig. 2A). The 7 others proteins whose expression increased over 1.5 were MARCO (3.27), PcgF5 (2.84), TRAF1 (2.50), Rilpl2 (2.46), Fas (2.28), C3 (2.25), MT1 (1.92), and GBP2 (1.81) respectively (Fig. 1B, labelled in blue). Of note, 4 of these 7 proteins are involved in inflammation (C3, MT1, TRAF1 and GBP2)^19–23^.

Next, we performed the proteomic analysis of non-infected versus infected primary macrophages extracted from mice bone marrow (BMMs). This analysis was challenged by the low amounts of cells that could be recovered, which lead to lower numbers of proteins identified, both from host and from bacteria. Up to 3019 host proteins were identified (3002 in non-infected cells and 2281 in infected cells, on average) and 121 *Francisella* proteins (in the infected samples) (Fig. 2B, Table S4). Nonetheless, supporting the data obtained in J774A.1, we found IRG1 was also upregulated in infected BMMs compared to non-infected BMMs. In BMMs, some proteins linked with interferon were identified in infected cells: Interferon regulatory factor 3 (IRF3), Interferon-induced helicase C domain-containing protein 1 (Ifih1) and Interferon-induced very large GTPase 1 (Gvin1). IRF-3 can be activated through TLR stimulation or through the cGAS/STING pathway. With IRF7, IRF3 is the major modulator of IFN gene expression. IRF3 is also known to activate PI3 K/Akt signaling needed to suppress proinflammatory genes and enhance anti-inflammatory genes^24^. Ifih1, also known as melanoma differentiation-associated protein 5 (MDA5), is a cytosolic RNA sensor belonging to the PRR family. It promotes innate and adaptive immune responses^25^. Gvin1 is regulated by IFNs in response to infection by intracellular bacteria. These data showed the recognition of pathogen and the ensuing immune response induced by macrophages that appears to be dominated by an IFN signature.

IRG1 was identified in J774A.1 macrophages and in BMM. It is known to play a central role during infection by various bacteria, including *Staphylococcus aureus, Brucella melitensis, Mycobacterium tuberculosis*, and *Coxiella burnetii*^26–29^. In particular, biochemical evidence established that itaconate production impaired the growth of bacteria in glucose-deprived conditions due to its ability to act as an inhibitor of the bacterial enzyme isocitrate lyase^30^. We therefore first wished to evaluate the possible role of the enzyme IRG1 (Fig. 2C) in *Francisella* entry (Fig. 2D) and intracellular multiplication (Fig. 2E, left panel). The J774A.1 macrophages transfected with siRNAs targeting IRG1 (siRNA IRG1) exhibited significant knockdown of Irg1 expression as demonstrated by qRT-PCR (Fig. 2C).

*Francisella* entry was only very mildly affected (89% of entry was recorded after 1 h infection in siRNA IRG1-transfected macrophages, compared to J774A.1 macrophages transfected with scrambled siRNA) (Fig. 2D). Intracellular bacterial multiplication, quantified by CFU counting at 10 h and 24 h (Fig. 2E), was not affected by IRG1 siRNA.

These data suggest that this protein, might play another role in *Francisella* infection and/or might be redundant actor in the network of proteins involved in response to *Francisella* infection.

### Cellular classes of proteins involved in *F. novicida* infection

**Figure 3:**
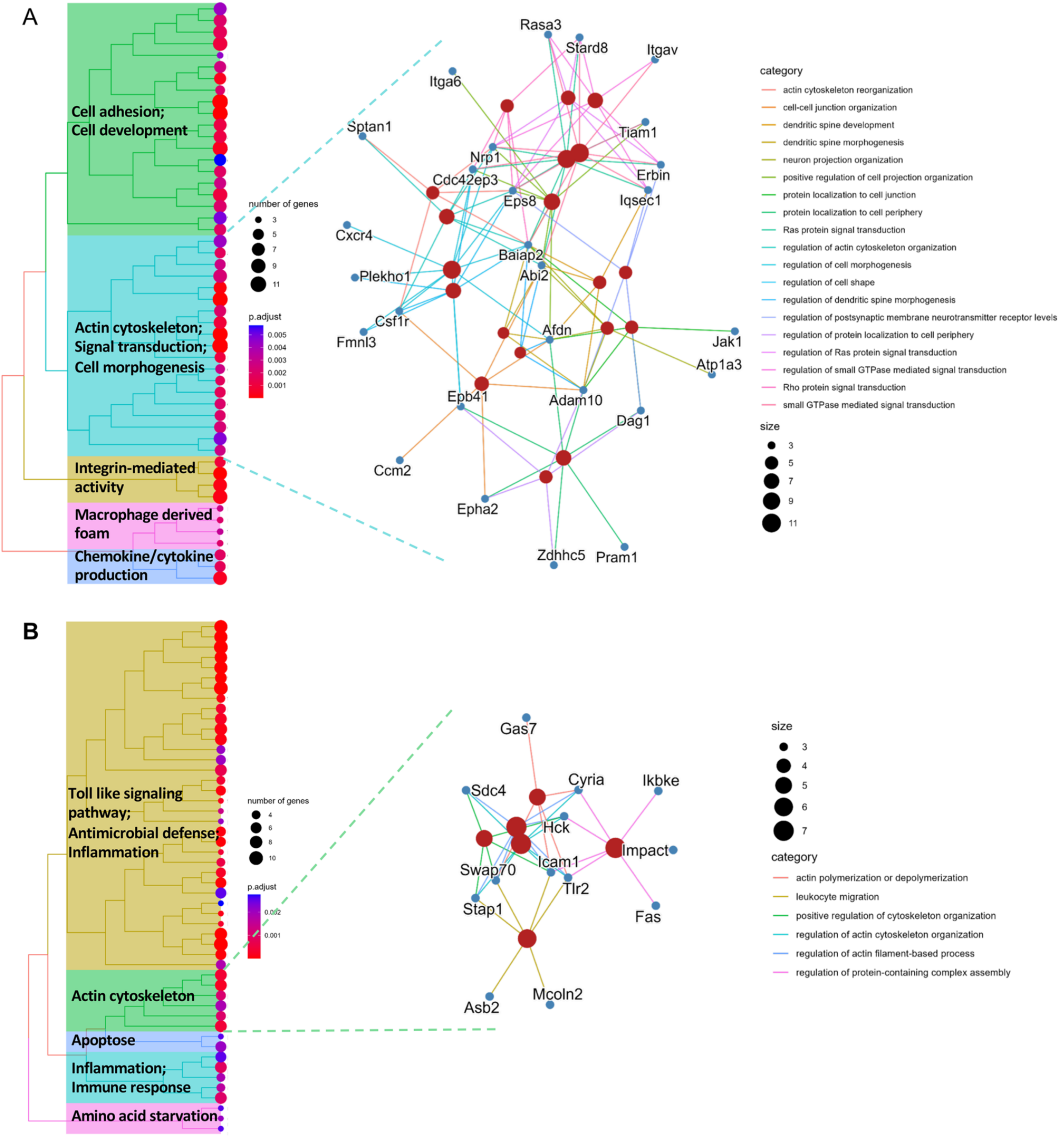
Enrichment of upregulated and downregulated proteins in infected macrophage compared to non-infected macrophages. (**A**) GO enrichment biological process performed on the differential macrophage downregulated proteins using ClusterProfiler, ranked according to adjusted p-value (here top50). (**B**) GO enrichment biological process performed on the differential macrophage upregulated proteins using ClusterProfiler, ranked according to adjusted p-value (here top50).

We next aimed to identify classes or complexes of proteins involved in *Francisella* infection. We conducted an enrichment analysis of upregulated and downregulated proteins during the infection from the *J774A.1* macrophages dataset as it was the most complete and robust (Fig. 3, Table S5). The downregulated proteins were enriched in protein related to motility such as “cell substrate adhesion” and “cell matrix adhesion” as well as proteins involved in “cell–cell junction” and “regulation of cell morphogenesis” (according to GO Biological Process annotation) (Fig. 3A). The upregulated proteins in infected macrophages were enriched in proteins related in antimicrobial defense, like “negative toll-like pattern receptor” and “actin cytoskeleton organization assembly” (according to GO Biological Process annotation) (Fig. 3B). Numerous proteins involved in actin regulation and regulation of cell morphogenesis were differentially expressed in infected macrophages in comparison with non-infected macrophages, and these two protein networks are closely interconnected (Fig. 3, Fig. S1).

In summary, these analyses indicate that after 10 h of infection, the macrophage response is characterized by a dual focus: one aspect on antimicrobial defense, and the other on the dynamic organization of the cytoskeleton.

### Dynamics of *Francisella* cell to cell spread

**Figure 4:**
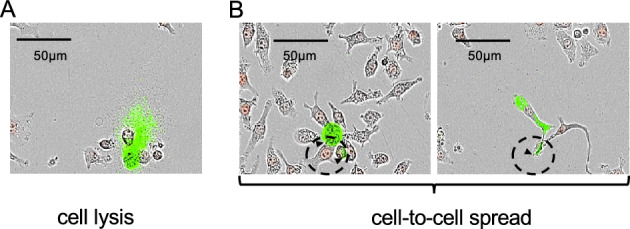
*Francisella* cell-to-cell dissemination in J774A.1 macrophages. Cells were infected for 48 h with wild-type *F. novicida* constitutively expressing plasmid-borne GFP (WT-GFP). Intracellular multiplication was followed by video-microscopy (20×). Different types of bacterial release from an infected cell are illustrated. (**A**) Pyroptosis: infected cells explode and release free bacteria in the extra-cellular medium; or (**B**,**C**) Cell-to-cell transfer (by cellular clustering, and “Kiss and run” spread, respectively).

Our proteomic and live cell imaging results suggested that after 10 h after infection, there is a morphological reorganization of macrophages when bacteria begin to propagate via merocytophagy, enabling their transmission through direct contact between an infected macrophage and one or multiple adjacent macrophages. We followed the dynamics of intracellular multiplication and dissemination of *F. novicida* in macrophages by time-lapse video microscopy, using a fully automated microscope (Incucyte^®^ 531 S3, Essen BioScience). J774A.1_red_ macrophages were infected with GFP-expressing *F. novicida* (WT-GFP) at a MOI of 100. Infection was then followed over a 48 h-period, in 96-well plates (Fig. 4, and Movies 1, 2, 3).

Overall, these analyses confirmed that *F. novicida* disseminate by either one two major routes: (i) by a necrotic form of cell death likely corresponding to pyroptosis, where cells filled with bacteria ultimately explode and release their bacterial content in the extracellular medium (Fig. 4A, Movie 1); and (ii) by direct cell-to-cell contact. In the latter case, we demonstrate for the first time, that two distinct types of contacts exist, designated “cellular clustering” and “kiss and run”. Indeed, either highly infected cells were first immobilized by multiple uninfected cells before cell-to-cell transfer occurred (“cellular clustering”, Fig. 4B left panel, Movie 2); or infected cells actively moved, established multiple contacts with several uninfected cells, before bacterial transfer could be visualized (“kiss and run”, Fig. 4B right panel, Movie 3). This observation is akin to the endosome maturation process^31^. In this case bacteria concentrated near the part of the cell membrane involved in the contact. Videos also show that multiple contact between infected cells and non-infected cells were needed to infect new cells. Moreover, we showed that, in J774.1 macrophages, infection of bacteria does not prevent replication of infected cells, resulting in two new infected cells that contribute to dissemination of *Francisella*.

### Cell-to-cell propagation requires actin polymerization

**Figure 5:**
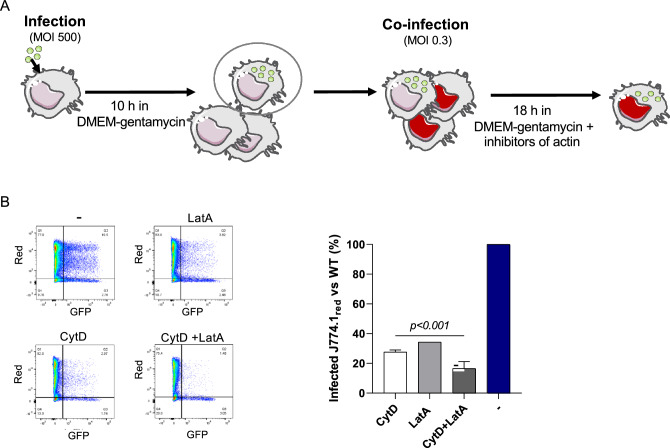
Cell-to-cell spread of *Francisella* requires actin polymerization. (**A**) Model of experiment: J774A.1 cells were infected for 10 h and then put on non-infected J774A.1_red_ cells in medium containing gentamicin and different inhibitors of actin polymerization for 18 h. P < 0.001 (as determined by ANOVA test). (**B**) The infected J774A.1_red_ cells (GFP-positive) were analyzed after 18 h of co-infection and graphical representation was presented by using Graphpad software.

Actin polymerization is essential for the realization of numerous cellular processes such as for example cell mobility and phagocytosis. To evaluate the role of actin polymerization in *Francisella* cell-to-cell transfer (here designated merocytophagy), we performed co-infections of J774A.1 macrophages with *Francisella*, in the presence or absence of two inhibitors of actin polymerization: Cytochalasin D (CytD) and Latrunculin A (LatA). Whereas CytD binds to the positive pole of actin filaments, LatA binds actin monomers. J774A.1 cells were infected with GFP-expressing WT *F. novicida*. At 10 h post-infection, these cells were then added onto uninfected J774A.1_red_ cells (expressing the red fluorescent protein mKate2) for 18 h, in medium supplemented with gentamicin to avoid extracellular infection. In this set-up, J774A.1_red_ infected cells (i.e. GFP-positive, mKate2-positive cells) correspond only to cells that became infected by direct cell to cell contact. Results were analyzed by using flow cytometry (Fig. 5A). We observed a severe reduction of cell-to-cell passage when either CytD or LatA was added (72,4%, and 65,7% decrease in the number of GFP-positive J774A.1_red_ cells upon treatment with CytD, and LatA, respectively). Furthermore, when both CytD and LatA were added, a synergistic inhibitory effect was recorded (84.6% reduction of cell-to-cell passage), demonstrating the contribution of actin polymerization to merocytophagy (Fig. 5B). Altogether these data suggest that response of macrophage to *Francisella* infection involved (i) cytoskeleton modification, (ii) a network of different actors that work together.

## Discussion

We combined here proteomics and cellular imaging to characterize the molecular mechanisms underlying *Francisella* infection in macrophages. Our proteomic analyses of infected macrophages defined a subset of bacterial proteins differentially expressed within the macrophage but also revealed the complex responses of the macrophage to the infection. Indeed, we showed that the majority of proteins that were expressed differently in infected macrophages compared to non-infected macrophages were related to antibacterial defense and cytoskeleton organization with the identification of numerous proteins involved in actin regulation. We also demonstrated that merocytophagy encompasses various type of cellular interactions and is dependent of actin polymerization.

Our data show that biotin biosynthesis may constitute the strongest bacterial adaptative response to the intracellular environment. This pathway is a key element for numerous pathogens such as *Pseudomonas aeruginosa* that is able to exploit it to benefit its infectivity^32^. In other bacteria such as *Mycobacterium tuberculosis*, BioA is involved in virulence in in the guinea pig model of tuberculosis^33^. Biotin synthesis is linked to virulence of *Francisella* and is for example, play a role in phagosomal escape of *Francisella*^12,14^. Of note, in a proteomic study, Twine et al. showed that bioB expression was decreased in *F. tularensis* LVS strain separated from infected murine spleen tissue in comparison with the same strain grown in broth^34^. These data suggest that biotin synthesis is finely regulated in *F. tularensis* and depend of the subsp. Indeed, *F. novicida* encodes two putative biotin protein ligase that have two distinct roles, one possesses the major ligase activity and was required for bacterial replication, whereas another one acts to regulate and thereby likely prevent wasteful synthesis of biotin^13^. In this study, we confirmed that biotin is not important for phagosomal escape but it is also involved during replication phase. We were not able to identify all the actors of positive regulation biotin synthesis suggesting that they were expressed before 10 h of infection and biotin were involved during a long time of intracellular phase. Moreover, these data raise the possibility of biotin synthesis as an anti-bacterial target^35^. Another protein that was over-expressed in infected macrophages is CspA that is thought to be linked to stress resistance. Indeed, CspA was shown to be involved in nisin resistance of *L. monocytogenes* and in acid resistance of *Brucella microti*^17,36^. The protein Slt (soluble lytic transglycosylase) was also upregulated in macrophages. This protein is involved in intracellular growth and immune suppression^18^. Altogether, these analyses indicate that *Francisella* express a panel of proteins that are implicated in its adaptation and survival in host cell.

Furthermore, our study revealed that throughout the cytosolic multiplication phase, a majority of the proteins encoded by the *Francisella* pathogenicity island (FPI) undergo downregulation within macrophages. Given the knowledge that FPI genes are up-regulated during active stringent response in the highly virulent *Francisella tularensis* subsp. *tularensis* SCHU S4, these findings suggest that *Francisella*'s adaptation to the macrophage cytosol varies depending on the subsp. In the case of *F. novicida* infection, the FPI-encoded T6SS, crucial for early phagosomal escape, seems dispensable for replication^15,16,37^. However, in remarkable study conducted by Kawula et al., it was shown that the *Francisella* type VI secretion system, encoded by FPI, is required for post-transfer endosomal escape after merocytophagy process, but not for cytosolic replication in the new cells^9^. Our data showed that FPI is not involved during replication phase. This observation suggests that the *F. novicida* cycle is intricately controlled, and the expression of virulence factors is contingent upon the timing of infection. Furthermore, various proteomic studies have revealed differences in protein expression depending on the *Francisella* subsp^38,39^. While such discrepancies in the proteomes of the *mediasiatica, tularensis*, and *holarctica* subsp may offer insights into the mechanisms employed by virulent strains of *F. tularensis,* a study employing two-dimensional electrophoresis compared the proteomes of representative strains from subsp *tularensis, holarctica*, and *mediasiatica.* The study identified 27 proteins that were either exclusive to, or expressed at least two fold greater abundance in subsp *tularensis*^38^.

In this study, we showed that most of bacterial proteins significantly upregulated and downregulated in macrophages are conserved across virulent *F. tularensis* subsps. Interestingly, only 6% of the identified proteins are exclusive to *F. novicida*, primarily associated with metabolic processes, ABC transporters, or with functions yet to be elucidated^41^. These findings suggest that *F. novicida* might employ shared mechanisms with its virulent counterparts in macrophages. However, further research is imperative to validate this hypothesis.

Numerous host proteins were upregulated upon infection with *Francisella*, ranging from immune-related proteins to metabolic enzymes, including notably IRG1, MARCO, Rilpl2, C3, PcgF5, MT1, TRAF1, GBP2 and Fas. The identification of IRG1 as main protein overexpressed during inflammation was expected. Indeed, IRG1 corresponds to the enzyme cis-aconitate decarboxylase that is responsible for the production of itaconate from the TCA derivative cis-aconitate. It exhibits a wide range immunoregulatory functions by limiting succinate dehydrogenase (SDH, being both the complex II of the respiratory chain and a TCA enzyme), blocking succinate-mediated inflammatory processes and inducing the anti-inflammatory proteins nuclear factor erythroid-related factor 2 (NRF2) and ATF3^42^. Moreover, the role of IRG1 as a host cell defense mechanism against *Francisella* infection has been reported^30^. Here, we showed that IRG1 was the most upregulated protein during *Francisella* infection. However, it had no direct impact on entry and intracellular multiplication of *Francisella*. Further researches are needed to understand the exact role of IRG1 during *Francisella* infection and to identify its potential co-actors. Of note one limitation of the current study is that the investigation of the potential antibacterial role IRG1 was performed in a macrophage cell line which may present a different metabolism from primary cells.

Our proteomic analysis has further substantiated the implication of certain proteins in *F. novicida* infection. Notably, GBP2, that we identified as overexpressed in J774.1 cells, has been recognized as a pivotal activator of AIM2 inflammasome both in vitro and in vivo^43^. A recent study showed that GBP2 recruitment to *F. novicida* is governed by the initial GBP1 recruitment^44^. Our analysis did not detect the presence of GBP1, implying that GBP1 might be recruited during earlier stages of infection. IFN regulatory factor 3 (IRF3), identified as overexpressed in BMM cells, has previously been associated with *Francisella* infection. IRF3 serves as a crucial transcriptional regulator of type I IFN-dependent immune responses and plays a pivotal role in AIM2 activation by *Francisella*^45^. Moreover, it has been reported that IRF3 protein expression is strongly dependent on SON. In line with these findings, our proteomic analysis revealed a notable overexpression of the SON protein in BMM^46^.

Global term analysis of the macrophage proteins deregulated during the early stages of dissemination allowed us to confirm acute inflammatory response. Inflammatory response varies depending on the *Francisella* subsp. Specifically, *F. novicida* triggers inflammasome activation, leading to the production of pro-inflammatory cytokines by infected macrophages^45,47,48^. In contrast, *F. tularensis* subsp. *tularensis* has the ability to evade inflammasome activation and suppress pro-inflammatory cytokine production^49,50^. Further investigations are necessary to elucidate the impact of *F. tularensis* subsp. *tularensis* on macrophages and the proteins involved in this process.

In addition, expression levels of proteins involved in cytoskeleton organization, cell–cell interaction and cell morphology were deeply altered in this stage. Morphology of cells are important for migration and here we show that migration of macrophages is involved in *F. novicida* dissemination. It has recently been shown that *Francisella* can disseminate by cell–cell contact without passing through the extracellular medium^51^. In this transient phenomenon called merocytophagy, bacterial transfer occurs from an infected cell to an uninfected phagocytic cell, leaving both the donor cell and the recipient cell intact and viable. Unlike what is observed with other intracellular bacteria with a cytosolic niche, no actin tail formation in *Francisella* to propel itself to adjacent cells was observed and the process is independent of autophagy^51^. In the recipient cell, bacteria initially reside in a double-membrane vacuole which requires a functional T6SS to promote bacterial access to the cytosolic compartment. Merocytophagy is an efficient bacterial transfer process that allows the simultaneous transfer of multiple bacteria compared to an infection with free living extra cellular bacteria where only one or two bacteria are generally internalized per infected cell^9^. It appears to be the major mode of dissemination used by *Francisella* and has been shown to be different from trogocytosis^9^. The recipient cells is always a phagocytic cells suggesting that phenomenon is relative to phagocytosis^51^. This hypothesis was enhanced by the enrichment that identify numerous proteins involved in actin regulation and cytoskeleton reorganization. In this study we demonstrate that in the presence of two inhibitors of actin polymerization, cell-to-cell dissemination of *Francisella* was severely compromised, indicating that cellular processes dependent on actin polymerization are involved, linking merocytophagy to phagocytosis. However, since donor cells are still alive after transfer, this transfer is distinct from a classical phagocytic process. The cellular actors involved in the actin-dependent process remains to be fully characterized. Merocytophagy might be both a host defense mechanism aimed to eliminate the pathogen and a pathogen-driven mechanism triggered to favor passage from cell to cell.

This is the first time that such depth in the proteomics of the bacterium and the host cell has been achieved. This is in part due to the pre-enrichment by FACS sorting of the infected macrophages and the level of their infection. Also, a crucial factor is the application of 4D proteomics, as it allows the necessary depth across the dynamic range of a dual proteomics (PMCID: PMC8453224). We believe that this new technology, linked to selective FACS sorting of infected cells can open the way to dual proteome analysis and cell –host interaction studies in the future.

Altogether, this study revealed the complexity of the macrophage response to *Francisella* infection. Further targeted analyses and validation studies (using defined media, inhibitors and inactivated cell lines) will enable further dissection of roles played by individual proteins and their pathways in regulating the behavior of *Francisella*-infected macrophages and their contribution to multiplication and merocytophagy.

## Methods

### Ethics statement

All Materials and Methods involving animals were conducted in accordance with ARRIVE guidelines and guidelines established by the French and European regulations for the care and use of laboratory animals (Decree 87-848, 2001-464, 2001-486 and 2001-131 and European Directive 2010/63/UE) and approved by the INSERM Ethics Committee (Authorization Number: 75-906, APAFIS # 24485]).

### Strains and culture conditions

Wild-type *F. novicida* (*F. tularensis* subsp. *novicida* strain U112) or *F. novicida* supplemented with a GFP carrying plasmid pKK-pGro-GFP^11^ were used. Plasmid pKK-pGro-GFP was introduced by chemical transformation into wild-type *F. novicida* to generate a strain constitutively expressing GFP (designated WT-GFP). Bacteria were grown at 37 °C on pre-made chocolate agar PolyViteX plates (BioMerieux), or Schaedler K3 medium.

### Cell cultures and cell infection experiments

J774A.1 (ATCC TIB-67™) cells or J774A.1 cells with red nuclei (J774A.1_red_) (expressing mKate2 nuclear-restricted red fluorescent protein) were propagated in Dulbecco’s Modified Eagle’s Medium (DMEM, PAA), containing 10% fetal bovine serum (FBS, PAA) unless otherwise stated. Preparation and culture of bone marrow derived macrophages (BMMs) were performed as previously described^52^. All mice were in the C57BL/6J background (Charles River, France). For CFU counting, the day before infection, approximately 2.10^5^ eukaryotic cells per well were seeded in 12-wells cell tissue plates and bacterial strains were grown overnight in 13 mL of Schaedler K3 at 37 °C. Infections were realized at a multiplicity of infection (MOI) of 100 for J774A.1 cells and incubated for 1 h at 37 °C in culture medium. After 3 washes with cellular culture medium, plates were incubated for 10 and 24 h in fresh medium supplemented with gentamicin (10 µg mL^−1^). At each kinetic point, cells were washed 3 times with culture medium and lysed by addition of 1 mL of distilled water for 10 min at 4 °C. Viable bacteria titers were determined by spreading preparations on chocolate agar PolyViteX plates. Each experiment was conducted at least twice in triplicates.

### Time lapse video microscopy

J774A.1_red_ cells were grown to confluence in 96-well cell tissue plates and were infected with GFP-expressing wild-type bacteria, at a MOI of 100. Plates were then incubated for 1 h at 37 °C in culture medium. After 3 washes with cellular culture medium, plates were incubated at 5% CO2 and 37 °C for 48 h in fresh medium with 5% of SVF supplemented with gentamicin (10 g mL^−1^). Bacterial multiplication was monitored in the fully automated microscope Incucyte^®^ S3 (Essen BioScience). Images were taken every 2 min with the 20× objective. Green and red fluorescence images were obtained every 2 min with an acquisition time of 400 ms and 200 ms respectively. Time-lapse videos (from which images were extracted) were generated by using Incucyte^®^ S3 and imageJ softwares.

### Proteomic analyses

#### Host cells

The day before infection, approximately 5.10^5^ J774A.1 macrophages per well were seeded in 6-wells cell tissue plates. Because of the lower infection capacity of *Francisella* in BMMs, 2.10^6^ BMM were seeded in T75 flasks. The bacterial strain U112-GFP were grown overnight in 13 mL of K3 at 37 °C. Infections were realized at a multiplicity of infection (MOI) of 500 for J774A.1 cells and BMMs and incubated for 1 h at 37 °C in culture medium. After 3 washes with PBS, plates were incubated for 10 h in fresh medium supplemented with gentamicin (10 μg mL^−1^). Infected cells were sorted and selected by using the FACS Sony MA900. The result was compared with a non-infected condition. Each sample was analyzed in five independent biological replicates. Protein concentration was determined by DC assay (Bio-Rad, CA) according to the manufacturer’s instructions.

#### Bacteria

Wild-type *F. novicida* U112 was grown overnight in 13 mL of K3 at 37 °C. Then, Bacteria were grown at 37 °C in K3 from OD 0.1 for 10 h. Bacteria were collected by centrifugation and the bacterial pellets were resuspended and lysed by sonication. Each strain was analyzed in three independent biological replicates. Protein concentration was determined by DC assay (Bio-Rad, CA) according to the manufacturer’s instructions.

#### Protein digestion

S-Trap™ micro spin column (ProtiFi, Huntington, USA) digestion was performed on bacterial lysates according to manufacturer’s instructions. Briefly, samples were reduced with 20 mM TCEP and alkylated with 50 mM CAA (chloracetamide) for 5 min at 95 °C. Aqueous phosphoric acid was then added to a final concentration of 2.5% following by the addition of S-Trap binding buffer (90% aqueous methanol, 100 mM TEAB, pH7.1). Mixtures were then loaded on S-Trap columns. Two extra washing steps were performed for thorough SDS elimination. Samples were digested with 1.5 µg of trypsin (Promega) at 47 °C for 2 h. After elution, peptides were finally vacuum-dried down.

#### NanoLC–MS/MS protein identification and quantification

The tryptic peptides were resuspended in 15 µL of 2% ACN, 0.1% FA in HPLC-grade water and a volume of 5 µL was injected on a nanoelute (Bruker Daltonics, Germany) HPLC (high-performance liquid chromatography) system coupled to a timsTOF Pro (Bruker Daltonics, Germany) mass spectrometer. HPLC separation (Solvent A: 0.1% formic acid in water; Solvent B: 0.1% formic acid in acetonitrile) was carried out at 200 nL/min using a packed emitter column (C18, 25 cm × 75 μm 1.6 μm) (IonOpticks, Australia) using a 70 min gradient elution (2–13% solvent B during 41 min; 13–20% during 23 min; 20–30% during 5 min; 30–85% for 5 min and finally 85% for 5 min to wash the column). Mass-spectrometric data were acquired using the parallel accumulation serial fragmentation (PASEF) acquisition method. The measurements were carried out over the m/z range from 100 to 1700 Th. The range of ion mobilities values from 0.75 to 1.25 V s/cm^2^ (1/k_0_). The total cycle time was set to 1.17 s and the number of PASEF MS/MS scans was set to 10. Ramp time was set to 100 ms. Target intensity and intensity threshold were set respectively to 15,000 and 500.

#### Data processing following nanoLC–MS/MS acquisition

The MS files were processed with the MaxQuant software version 2.0.1.0 and searched with Andromeda search engine against the UniProtKB/Swiss-Prot and TrEMBL *F. novicida* database (release 2021, 2182 entries) and the UniProtKB/Swiss-Prot *Mus Musculus* database (release 11-2021, 17089 entries). To search parent mass and fragment ions, we set a mass deviation of 10 and 40 ppm respectively. The minimum peptide length was set to 7 amino acids and strict specificity for trypsin cleavage was required, allowing up to two missed cleavage sites. Carbamidomethylation (Cys) was set as fixed modification, whereas oxidation (Met) and N-term acetylation were set as variable modifications. The false discovery rates at the protein and peptide levels were set to 1%. Scores were calculated in MaxQuant as described previously. The reverse and common contaminants hits were removed from MaxQuant output. Proteins were quantified according to the MaxQuant label-free algorithm using LFQ intensities; protein quantification was obtained using at least 1 peptide per protein.

Statistical and bioinformatic analysis, including heatmaps, profile plots, normalisation and clustering, were performed with Perseus software (version 1.6.15.0) freely available at http://www.perseus-framework.org while the correlation plots were performed by R. For statistical comparison in the infected macrophages analysis, we set two groups, infected (I) and non-infected (NI) each containing five biological replicates. We then filtered the data to keep only proteins with all 5 valid values out in at least one group. Next, the data were imputed to fill missing data points by creating a Gaussian distribution of random numbers with a SD of 33% relative to the SD of the measured values and 1.8 SD downshift of the mean to simulate the distribution of low signal values. We performed *t* test, using FDR < 0.01, S0 = 0.1. To better explore protein changes in the mouse proteome, because of the low yield of infected BMM cells, bacteria proteins were excluded from the matrix and the LFQ intensities were further normalized by width adjustment to account for the unbalance induce buy the presence of the bacterial proteome and the lower number host proteins identified in infected cells. Briefly, the first, second and third quartile (q1, q2, q3) are calculated from the distribution of all values. The second quartile (which is the median) is subtracted from each value to center the distribution. Then we divide by the width in an asymmetric way. All values that are positive after subtraction of the median are divided by q3–q2 while all negative values are divided by q2–q1 (http://www.coxdocs.org/doku.php?id=perseus:user:activities:matrixprocessing:normalization:widthadjustment). Finally, we performed *t* test on the mouse dataset as well, using FDR < 0.01, S0 = 0.1.

Volcano plot performed with Perseus with the same threshold were customized using ggplot2 R package from tidyverse (version 2.0) on R/Rstudio (version 4.2.2/version 2022.07.2).

Gene Ontology Over Representation Analysis (GO ORA) was performed on the significant up- and down-regulated proteins using ClusterProfiler R package (version 4.6.2) for the GO ORA analysis and org.Mm.eg.db R package (version 3.16.0) as a genome wide annotation for human using following function arguments (ont = "BP", OrgDb = "org.Mm.eg.db", pAdjustMethod = "BH", pvalueCutoff = 0.05, qvalueCutoff = 0.05). All the genes listed in the database were used as background (“universe” argument by default). Finally, enrichplot R package (version 1.18.4) was used for data visualization (network plot, treeplot).

### RNAi

J774A.1 macrophages were transfected with 100 pM siRNA targeting IRG1 (PDSIRNA5D, Merck), or siRNA Negative Control Med GC (Life Technologies), using Lipofectamine RNAiMAX (Life Technologies) according to the manufacturer's instructions. Transfection complexes were removed one day and a half after transfection.

### RNA isolation, reverse transcription and quantitative PCR

Total RNA was isolated from cells using the RNeasy Plus Mini Kit (QIAGEN) following the manufacturer’s instructions. RNA concentrations were determined using a Nanodrop system. 250 ng of RNA reverse transcribed using the LunaScript RT SuperMix Kit (NEB) according to the manufacturer's instructions. qPCR was performed using the Luna Universal qPCR Master Mix Kit (NEB), and the following cycler program: 95 °C for 1 min, 40 cycles at 95 °C for 15 s, and 60 °C for 30. Each point was performed in technical triplicate. The relative abundance of IRG1 mRNA (sense: 5′-AAACGTTGGCTTCCATCCCAT-3′; antisense: 5′-CCAAAGAGATTCCACCCTCCC-3′) was calculated by the comparative ΔΔCt method normalizing to the housekeeping gene product GAPDH mRNA (sense: 5′-TGCACCACCAACTGCTTA-3′; antisense: 5′-GGATGCAGGGATGTTC-3′) and comparing to one reference sample indicated on each graph. The results are presented as an n-fold difference relative to reference sample.

### Cell sorting and flow cytometry

WT-GFP *F. novicida* was grown overnight in 13 mL of Shaedler K3 medium at 37 °C. Infections were realized at a multiplicity of infection (MOI) of 500 and cells were incubated for 1 h at 37 °C in culture medium. After 3 washes with cellular culture medium, plates were incubated for 10 h in fresh medium supplemented with gentamicin (10 µg mL^−1^). Cells were washed once with PBS supplemented with gentamicin. Infected cells were sorted and selected by using the FACS Sony MA900. Infected cells were put on J774A.1 macrophages with red nuclei (J774A.1_red_) with a MOI of 0,3. Cells were incubated for 18 h in fresh medium supplemented with gentamicin (10 µg mL^−1^) at 37 °C. Cells were washed twice with PBS, supplemented with gentamicin (10 µg mL^−1^), scratched, and fixed in 4% PFA. Cells were analyzed by using the BD LSR Fortessa. The collected data were processed with CytExpert software (Beckman Coulter) and presented using FlowJo (FlowJo LLC, Ashland, OR, USA).

### Statistics

In vitro experiments were at least repeated twice and in triplicates. Data were analyzed using GraphPad Prism software. Tests are specified in each legend. In figures, all the results correspond to mean ± SEM.

### Supplementary Information

Supplementary Figure S1.

Supplementary Video 1.

Supplementary Video 2.

Supplementary Video 3.

Supplementary Legends.

Supplementary Legends.

Supplementary Legends.

Supplementary Table S1.

Supplementary Table S2.

Supplementary Table S3.

Supplementary Table S4.

Supplementary Table S5.

## Data Availability

The mass spectrometry proteomics data have been deposited to the ProteomeXchange Consortium via the PRIDE [1] partner repository with the dataset identifier PXD035145 and 10.6019/PXD035145. The authors declare that all other data supporting the findings of this study are available within the paper and its Supplementary Information files.

## Abbreviations

WT: *Francisella novicida* U112
BMM: Bone marrow-derived mouse macrophages
CFU: Colony forming unit
DMEM: Dulbecco’s modified eagle medium
IRG1: Immunoresponsive gene 1
Subsp: Subspecies
KEGG: Kyoto encyclopedia of genes and genomes
LC–MS/MS: Liquid chromatography coupled to tandem mass spectrometry
LFQ: Label free quantification
FDR: False discovery rate
MOI: Multiplicity of infection
OD: Optical density
