# Supplementary material for: Dual proteomics of infected macrophages reveal bacterial and host players involved in the Francisella intracellular life cycle and cell to cell dissemination by merocytophagy
Source: Sci Rep. 2024 Apr 2;14:7797. doi: 10.1038/s41598-024-58261-x (PMC10987565; doi:10.1038/s41598-024-58261-x)
Supplement: Supplementary file 1 — Supplementary Figure S1. [file 41598_2024_58261_MOESM1_ESM.pdf]

A

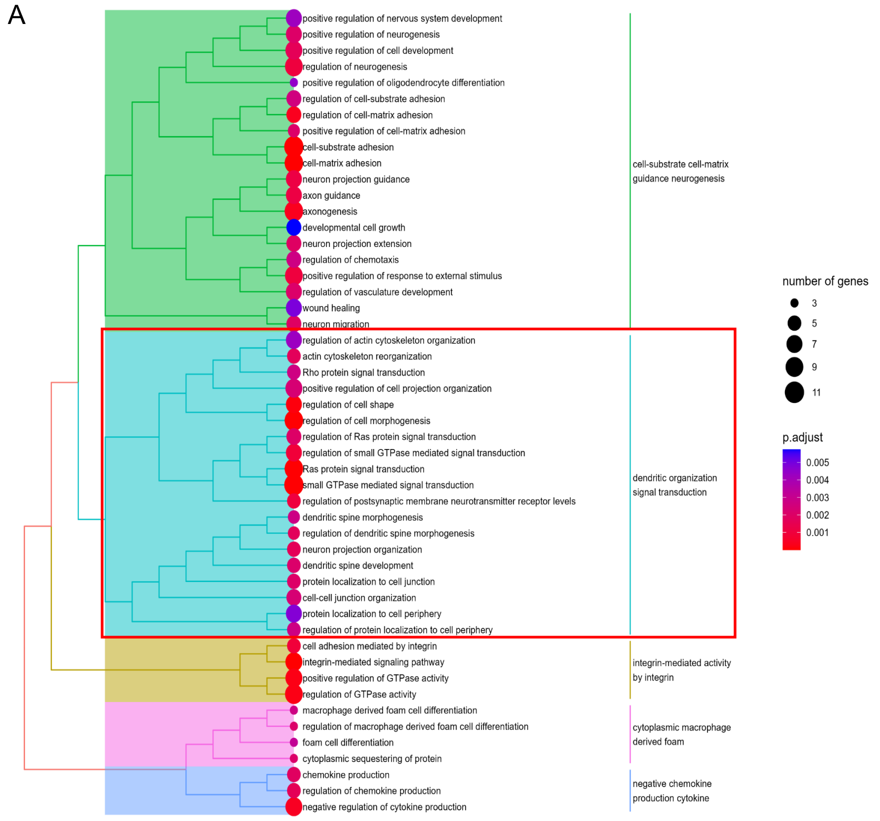

Down-regulated host (macrophage) proteins (Biological Process) Treeplot (top50)

B

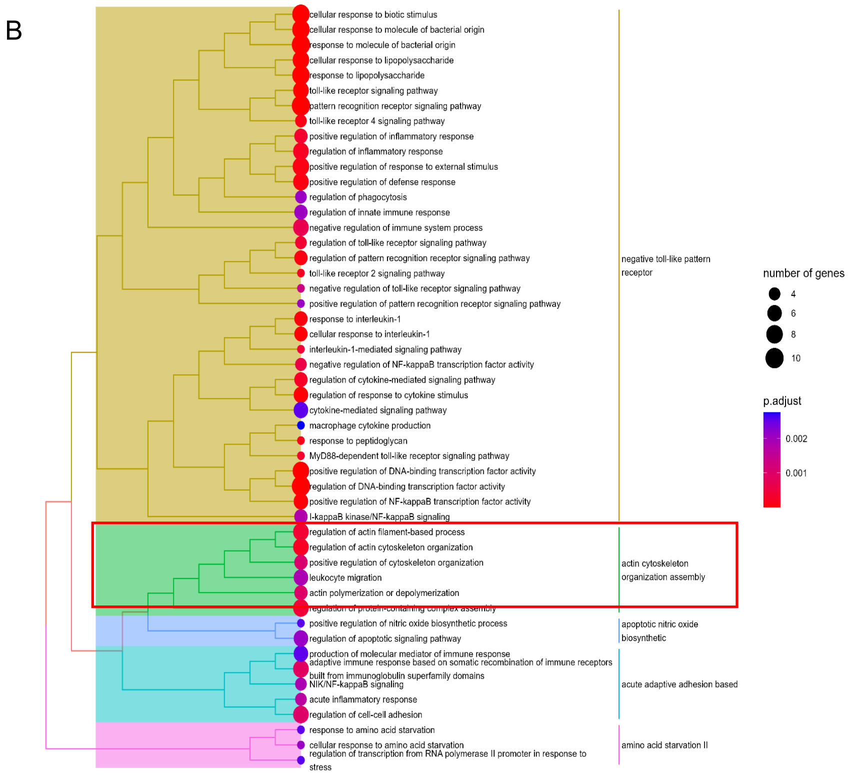

Up-regulated host (macrophage) proteins (Biological Process) Treeplot (top50)

Figure S1 Enrichment of upregulated and downregulated proteins in infected macrophage compared to non-infected macrophages

(A) GO enrichment biological process performed on the differential macrophage downregulated proteins using ClusterProfiler, ranked according to adjusted p-value (here top50). (B) GO enrichment biological process performed on the differential macrophage upregulated proteins using ClusterProfiler, ranked according to adjusted p-value (here top50).
