# Supplementary material for: Dual proteomics of infected macrophages reveal bacterial and host players involved in the Francisella intracellular life cycle and cell to cell dissemination by merocytophagy
Source: Sci Rep. 2024 Apr 2;14:7797. doi: 10.1038/s41598-024-58261-x (PMC10987565; doi:10.1038/s41598-024-58261-x)
Supplement: Supplementary file 7 — Supplementary Legends. [file 41598_2024_58261_MOESM7_ESM.docx]

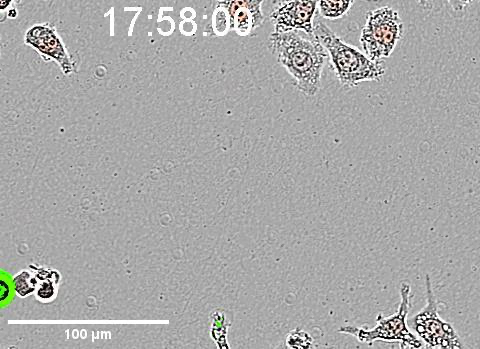
**Video S3. Cell to cell dissemination** **(2): Kiss and run**:  J774.1 macrophages were infected in DMEM-Glucose at an MOI of 100 with wild-type *F. novicida*expressing pKK214::pGrogfp (designated WT-GFP). The infected cell moves and gets in contact with several uninfected cells. After numerous contacts with the same uninfected cell, cell-to-cell transfer of *Francisella* occurs.
